# The Lid Domain of *Caenorhabditis elegans* Hsc70 Influences ATP Turnover, Cofactor Binding and Protein Folding Activity

**DOI:** 10.1371/journal.pone.0033980

**Published:** 2012-03-29

**Authors:** Li Sun, Franziska T. Edelmann, Christoph J. O. Kaiser, Katharina Papsdorf, Andreas M. Gaiser, Klaus Richter

**Affiliations:** Center for Integrated Protein Science Munich (CIPSM) and Department Chemie, Technische Universität München, Garching, Germany; University Medical Center Groningen, University of Groningen, The Netherlands

## Abstract

Hsc70 is a conserved ATP-dependent molecular chaperone, which utilizes the energy of ATP hydrolysis to alter the folding state of its client proteins. In contrast to the Hsc70 systems of bacteria, yeast and humans, the Hsc70 system of *C. elegans* (CeHsc70) has not been studied to date.

We find that CeHsc70 is characterized by a high ATP turnover rate and limited by post-hydrolysis nucleotide exchange. This rate-limiting step is defined by the helical lid domain at the C-terminus. A certain truncation in this domain (CeHsc70-Δ545) reduces the turnover rate and renders the hydrolysis step rate-limiting. The helical lid domain also affects cofactor affinities as the lidless mutant CeHsc70-Δ512 binds more strongly to DNJ-13, forming large protein complexes in the presence of ATP. Despite preserving the ability to hydrolyze ATP and interact with its cofactors DNJ-13 and BAG-1, the truncation of the helical lid domain leads to the loss of all protein folding activity, highlighting the requirement of this domain for the functionality of the nematode's Hsc70 protein.

## Introduction

Hsc70 and its heat-shock inducible homolog Hsp70 are ATP-dependent molecular chaperones which bind unfolded proteins [1]. They participate in various cellular processes as diverse as protein *de novo* folding, protein translocation across organelle membranes and uncoating of clathrin-coated vesicles [2]–[8]. In eukaryotes, several cytosolic variants of Hsp70-like proteins with distinct features are encoded. Some, like the yeast proteins Ssb1, Ssb2 and Ssz1, reside at the ribosome as part of the ribosome-associated complex (RAC), while others, such as Hsc70s and the heat-inducible Hsp70s are assumed to be diffusible factors in the cytosol. Two Hsc70-homologs (Ssa1 and Ssa2) are expressed in budding yeast at normal growth conditions and two Hsp70s (Ssa3 and Ssa4) are expressed only in response to stress. The simultaneous knockout of *SSA1* and *SSA2* is lethal at elevated temperatures [9], but the general redundancy of Hsp70/Hsc70-proteins complicates analysis *in vivo*. While the mammalian system is even more complex [10], in *C. elegans* only one Hsc70-like protein, HSP-1, exists (termed CeHsc70 here) and its three Hsp70-proteins (HSP-70, F44E5.4, F44E5.5) are only expressed in response to heat-shock [11], [12]. The RNAi-mediated knockdown of CeHsc70 has dramatic consequences, leading to increased protein aggregation [13] and arrested development at early larval stages [14], [15], confirming that essential and non-redundant cellular functions are performed by this homolog of Hsc70.

Hsc70 chaperones generally are arranged in three domains: an N-terminal nucleotide binding domain (NBD), a substrate binding middle domain (SBD), and a C-terminal helical domain, which covers the substrate binding groove of the SBD [16], [17]. While the helical lid domain diverges strongly between eukaryotic and prokaryotic species, the NBD and SBD are highly conserved. Biochemical studies of the bacterial Hsp70-protein DnaK described many aspects of the ATP-hydrolysis mechanism and defined a hydrolysis cycle, which is coupled to the substrate processing activity: An ATP-bound state of Hsp70 binds substrates weakly. After ATP hydrolysis, the substrate is efficiently bound by ADP-Hsp70. This complex is resolved slowly by the release of ADP and substrate (reviewed in [3], [4]). All Hsp70 domains are supposedly participating in and communicating during this process [18]–[20]. While it was shown that the helical lid domain covers the substrate binding groove of the SBD [21] and is important for efficient protein folding [22] the mechanistic features of its involvement are not fully understood yet.

Two distinct types of cofactors influence the ATPase cycle in all species (reviewed in [23], [24]). J-domain containing proteins, like mammalian Hsp40s or bacterial DnaJ, accelerate the hydrolysis reaction of Hsp70s [25]. Nucleotide exchange factors (NEFs), like bacterial GrpE or human Bag1, specifically facilitate the release of the nucleotide after hydrolysis [26]–[28]. The combined action of these proteins strongly accelerates ATP turnover of Hsp70 proteins [27], [29]. This acceleration has been observed for both, the bacterial system, composed of DnaK, DnaJ and GrpE [27], [28], [30]–[32] as well as the eukaryotic system, consisting of Hsp70, Hsp40 and Bag1 [29]. For bacteria, the full system of DnaK, DnaJ and GrpE is required to efficiently refold substrate proteins [30], [33], [34]. Contrarily, in eukaryotes the participation of Bag1 and other NEFs in the folding process has been reported to be paradoxically both: unfavorable [35], [36] or supportive [37], [38].

Despite its importance as a model of genetics and developmental biology, the Hsc70 system of *Caenorhabditis elegans* has not been analyzed *in vitro* to date. Using bioinformatics, the encoded Hsp70-like proteins can be assigned to the various compartments they work in [39]: One mitochondrial Hsp70-protein (HSP-6), two ER-based homologs (HSP-3 and HSP-4) and one ribosomally attached Hsp70-protein (F11F1.1) exist in addition to the cytosolic Hsc70/Hsp70 proteins mentioned before. For the sole and essential CeHsc70 protein only few studies provide biochemical and structural data [14], [40]. With BAG-1, the CeHsc70 system features a shortened, distantly related, non-essential homologue of human Bag1 [41], [42]. One Sis1 (or DNAJB) homolog can be found in C. *elegans*: DNJ-13. It appears to be essential [42]. In this study, we address the biochemical characteristics of nematodal Hsc70 and its cofactors DNJ-13 and BAG-1. In this context, we also investigate the contribution of the helical lid to the regulation of the high turnover rate and the rate-limiting step of the CeHsc70 ATPase, the protein's affinity towards cofactors, and its ability to refold proteins.

## Results

### The high ATPase activity of CeHsc70 is limited by nucleotide release

**Figure 1:**
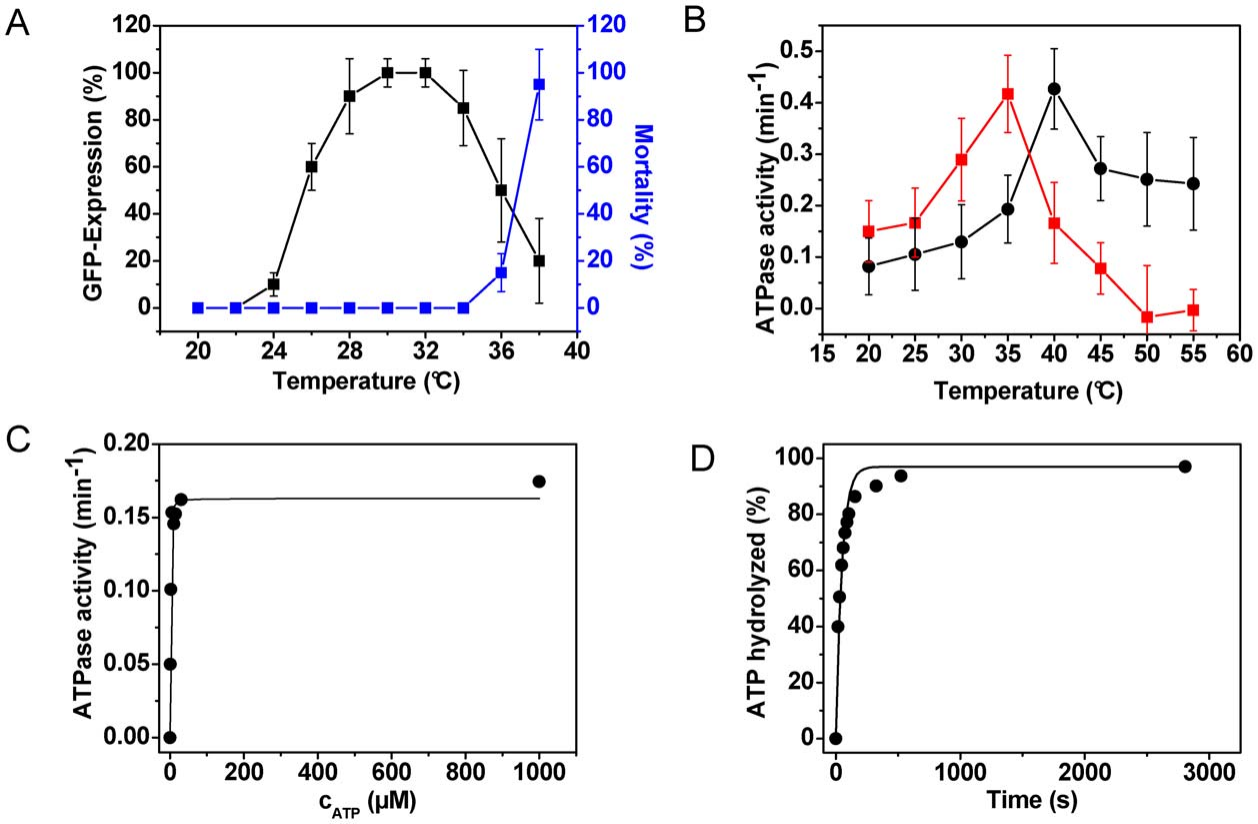
Characterization of CeHsc70. (**A**) The induction of the heat-shock response (black squares, left ordinate) was analyzed by exposing a *hsp-70*::GFP containing *C. elegans* strain to different temperatures for two hours and scoring after a recovery time of twelve hours. For this experiment nematodes at YA stage were used. The worms were grown at 20°C before shifting them to the respective heat-shock temperature. The percentage of mortality in percent of deceased animals (blue squares, right ordinate) was determined from these samples as well. The values presented are an average of three independent experiments and the error bars represent the standard deviation. (**B**) The dependence of the ATPase rate of CeHsc70 (red squares) and HsHsc70 (black circles) was determined under steady-state conditions as described in the Materials and Methods section. The values represent the mean of three replicates with the corresponding standard deviation given as errors. (**C**) Determination of the K_M_-value of CeHsc70 (•) for ATP in standard buffer at 25°C. Steady-state ATPase activities were determined for CeHsc70 at different ATP concentrations. The data were analyzed as described in the Materials and Methods section. (**D**) Single-turnover measurement of 20 µM CeHsc70 (•) in the presence of 4 µM ATP in standard buffer at 25°C. Data were analyzed as described in the Materials and Methods section.

We purified recombinant His_6_-CeHsc70 (referred to as CeHsc70 throughout the manuscript) and studied the ATPase cycle by a combination of steady-state and single-turnover experiments. Using an ATP-regenerating system we determined a k_cat_ of 0.18 min^−1^ for the steady-state hydrolysis rate at 25°C (Table 1). This is higher than values reported for the bacterial, yeast and mammalian proteins, which hydrolyze ATP at turnover rates of 0.05 min^−1^, 0.01 min^−1^ and 0.1 min^−1^, respectively, at 30°C (Table 1 and [4], [37], [43]–[46]). This temperature is well above the optimal growth temperature of *C. elegans* and already in a range, where Hsp70 induction is strong as a part of the general heat-shock response in this organism (Figure 1A). In fact, the nematodal Hsc70 starts to unfold at 34°C (Figure S1). To study the above mentioned divergence in activity between the *C. elegans* and human protein (HsHsc70) more closely, we assessed the temperature dependence of the ATPase activity. Surprisingly, the optimum of the ATPase rate of both proteins coincides with temperatures, considered lethal for both organisms (Figure 1B). Furthermore, both Hsc70 orthologs are - in a nucleotide-bound state - still stably folded at these temperatures (Figure S1). We determined the K_M_-value of CeHsc70 to be <3 µM (Figure 1C). In order to determine the rate-limiting step of the ATPase reaction catalyzed by CeHsc70, we performed single-turnover experiments. In these experiments we used substoichiometric concentrations of ATP to determine the rate of the first hydrolysis step. Under single-turnover conditions CeHsc70 hydrolyzed ATP at a rate of 1.29 min^−1^±0.18 min^−1^ (Table 1, Figure 1D). This rate is ∼8-fold higher than the steady-state hydrolysis rate, which implies that the hydrolysis cycle of the nematodal Hsc70 protein is limited by the release of the ADP-molecule after the hydrolysis reaction. It also shows that the nematode's protein differs from many other Hsp70 chaperones analyzed before, which are mostly limited by ATP hydrolysis (Table 1 and [4]), suggesting a certain diversity in the enzymatic mechanism of Hsp70 proteins, despite the high level of sequence conservation.

**Table 1:** Biophysical and enzymatic characterization of lid domain mutants.

	T_M_ DSF	T_M_ CD	K_M_	k_cat_	k_hyd_
CeHsc70	39°C	38°C	tight	0.18±0.04 min^−1^	1.29±0.18 min^−1^
CeHsc70-Δ384	39°C	38°C	tight	0.21±0.04 min^−1^	0.32±0.05 min^−1^
CeHsc70-Δ512	39°C	41°C	tight	0.14±0.02 min^−1^	1.03±0.41 min^−1^
CeHsc70-Δ545	39°C	37°C	tight	0.09±0.02 min^−1^	0.08±0.02 min^−1^

ATPase activities were determined in standard buffer as described in the Materials and Methods section. The K_M_-determination was carried out at 2 µM protein concentration and curves showed very tight binding and full saturation at stoichiometric concentrations, implying that the K_M_ value is smaller than or around 2 µM. Consequently, Michaelis-Menten conditions are not maintained and a determination of an apparent K_D_ value is not permitted by this experimental setup (indicated by “tight”). K_D_ denotes the apparent affinity. The errors represent standard deviations of three independent experiments.

### Truncations in the lid domain alter the rate-limiting step of the hydrolysis cycle

**Figure 2:**
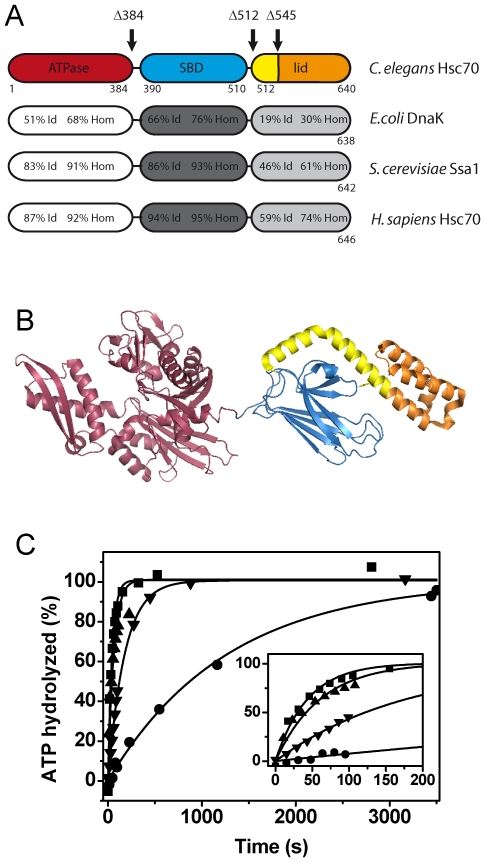
CeHsc70 truncation mutants show an altered ATP turnover. (**A**) Domain organization and amino acid identity (Id) and homology (Hom) of CeHsc70 towards bacterial, yeast and human homologs. The truncation mutants generated in this work are indicated by black arrows. (**B**) Structure of DnaK based on the PDB file 2KHO [93]. The truncations are colored in red (CeHsc70-Δ384), red and blue (CeHsc70-Δ512) and red, blue and yellow (CeHsc70-Δ545). The lid region, which is missing in the CeHsc70-Δ545 mutant, is highlighted in orange. (**C**) The single-turnover experiments using 20 µM CeHsc70 variants were performed as outlined in the Materials and Methods section in standard buffer at 25°C. Data for CeHsc70-Δ384 (▾), CeHsc70-Δ512 (▴), CeHsc70-Δ545 (•) and CeHsc70 (▪) were fit to single exponential functions. The inset shows the initial phase of the hydrolysis reaction within the first 200 s.

In order to understand which domains of CeHsc70 are responsible for the enzymatic activity, we generated C-terminal deletion fragments. As removal of the His_6_-tag from our protein only had minor impact on the ATPase rate (Figure S2), we designed the fragments accordingly and continued to work with the His_6_-tagged versions. While the overall amino acid sequence of CeHsc70 is strongly conserved, a high diversity can be found in the helical lid domain at the C-terminus (Figure 2A) [47]. Very little similarity is detectable between bacterial and metazoan Hsc70 proteins in this stretch of 130 amino acids. We generated fragments, which lack the whole substrate binding domain (CeHsc70-Δ384) or the C-terminal lid structure (CeHsc70-Δ512). Additionally, a fragment was created, lacking the very C-terminal helix bundle of the lid domain (CeHsc70-Δ545) retaining only helix A and half of helix B (Figure 2A and 2B) to avoid the generation of artificial hydrophobic interaction surfaces. We purified these fragments and confirmed that their tertiary structure was uncompromised by limited proteolytic digestion and thermal denaturation detected by circular dichroism (CD) and differential scanning fluorimetry (DSF). CD thermal transitions indicated the unfolding midpoint of secondary structure elements for all fragments to be in the range of 37–41°C (Figure S3A, Table 1). Limited proteolysis also confirmed that the overall stability of the core protein was unaltered by the truncations (Figure S3B). DSF further stressed that the fragments are not destabilized compared to the full-length protein, all having a transition midpoint at 38°C (Figure S3C and S3D, Table 1). We also aimed at understanding the influence of nucleotides on the stability of the full-length protein and the fragments. We thus recorded DSF transitions in the presence of ADP and observed a shift of about 10°C in the transition midpoint of nematode and human Hsc70 (Figure S2). The same shift also was observable when using the truncation fragments Δ545, Δ512 (Figure S3C) and Δ384 (Figure S3D), implying that neither the stability of the fragments nor the ability to bind nucleotides is compromised by the C-terminal truncations, which is also implied the very tight binding observed in steady-state assays (Table 1). We further determined the ATPase activities of the truncation fragments. The steady-state turnover rates were only slightly affected: The isolated ATPase domain hydrolyzed ATP with a k_cat_ of 0.21 min^−1^. CeHsc70-Δ512 and CeHsc70-Δ545 exhibited reduced ATPase activities of 0.14 min^−1^ and 0.09 min^−1^, respectively (Table 1).

In order to determine the rate-limiting step of these variants, single-turnover experiments were performed. Here, the isolated ATPase domain, CeHsc70-Δ384, exhibited a single-turnover hydrolysis rate of 0.32 min^−1^ (Figure 2C, Table 1). The similarity of steady-state and single-turnover rates suggests that the hydrolysis of the ATP molecule has become rate-limiting for this truncation variant. In contrast, a faster single-turnover hydrolysis rate of 1.03 min^−1^ was detected for CeHsc70-Δ512, suggesting that sequences C-terminal of the ATPase domain accelerate the hydrolysis reaction and shift the rate-limiting step towards nucleotide release (Figure 2C, Table 1). Surprisingly, the presence of half the lid domain in CeHsc70-Δ545 resulted in a protein with dramatically slower single-turnover kinetics (0.08 min^−1^, Figure 2C, Table 1). This reaction is much slower than the single-turnover rate of the full-length protein and matches the steady-state turnover rate for this mutant. The concurrence of steady-state and single-turnover rates demonstrates that for CeHsc70-Δ545 the rate-limiting step is prior to or during the hydrolysis reaction. Based on these results, the helical lid domain of CeHsc70 has the potential to influence the rate-limiting step of the ATPase cycle and shift it between hydrolysis steps and nucleotide release.

### The NEF-function of nematodal BAG-1 is conserved for all truncation fragments

**Figure 3:**
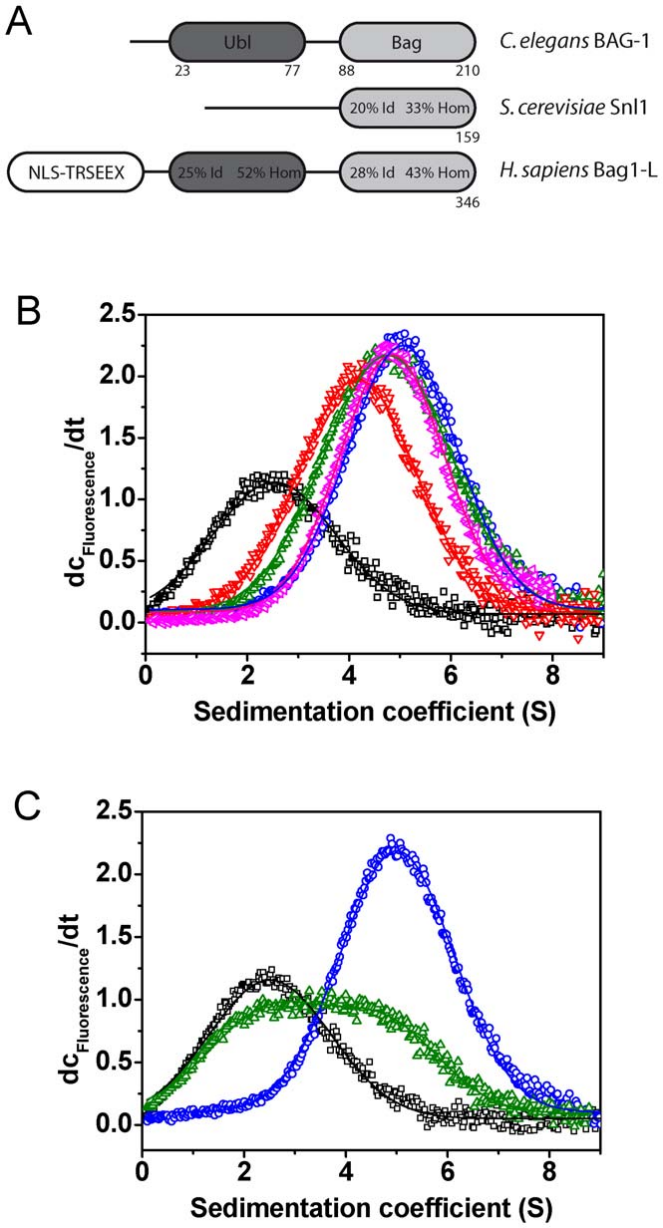
The function of the NEF BAG-1 is conserved in context with CeHsc70. (**A**) Domain organization of BAG-1 homologs from *C. elegans*, *H. sapiens* and *S. cerevisiae*. Percentages relate to identical (Id) and homolog (Hom) residues in respect to the nematode protein (Ubl: ubiquitin-like domain, Bag: BAG-domain, NLS: nuclear localization signal, TRSEEX: region containing multiple repetitions of the pentapeptide TRSEEX. (**B**) dc/dt plots were generated from sedimentation velocity experiments of 300 nM *BAG-1 in the absence (black) or presence of 3 µM CeHsc70 (blue) or 3 µM of the isolated ATPase domain CeHsc70-Δ384 (red), the full lid-deletion CeHsc70-Δ512 (green) and the half-lid deletion construct CeHsc70-Δ545 (pink). (**C**) dc/dt plots were generated from sedimentation velocity experiments of 300 nM *BAG-1 alone (black) or in the presence of 3 µM CeHsc70 (blue). The influence of nucleotides was analyzed by addition of 4 mM ATP (green) to 300 nM *BAG-1 and 3 µM CeHsc70. Data were analyzed as described in the Materials and Methods section.

As truncations in the lid domain lead to alterations to the rate-limiting step of the CeHsc70 ATPase cycle, it is also interesting to learn how these alterations influence cofactor interactions. The dominant NEF for CeHsc70, BAG-1, is weakly conserved in *C. elegans* (Figure 3A), with the human protein being only 43% homologous within the BAG-domain and the N-terminal domain being absent. The BAG-domain binds to the NBD of Hsc70 and induces a conformation unable to bind nucleotide [48]. A previous structural study of the isolated nematodal BAG-domain revealed that this protein is potentially dimeric and that the binding site for CeHsc70 is structurally altered compared to human Bag1 [41].

We purified full-length BAG-1 and tested its interaction with CeHsc70 and fragments of CeHsc70 by employing analytical ultracentrifugation (AUC). To this aim, we labeled BAG-1 at its internal cysteine residue Cys7 (*BAG-1) and performed sedimentation experiments in the absence and presence of CeHsc70. *BAG-1 sedimented with a sedimentation coefficient of 2.1 S and characteristics of a monomeric protein (s_20,w_ = 2.1±0.4 S; D_20,w_ = 7.36*10^−7^±1.5*10^−7^ m^2^ s^−1^; MW∼23.5 kDa). While this does not support the dimeric structure, which was proposed based on the crystal contact interfaces [41], the monomeric nature matches earlier studies on mammalian Bag1 [49]. Binding to CeHsc70 was strong and resulted in a protein complex at 4.8 S (Figure 3B). This value is somewhat larger than the s_20,w_-value of monomeric CeHsc70, which is 4.3 S (own data and [50] for human Hsc70). Binding to *BAG-1 was also observed for the other variants of CeHsc70 (Figure 3B).

We further utilized this AUC assay to determine the influence of nucleotides on the binding between *BAG-1 and CeHsc70. In the presence of 4 mM ATP, the *BAG-1•CeHsc70 interaction was strongly suppressed. Only 22% of *BAG-1 bound to CeHsc70 compared to the nucleotide free set-up, where >85% of *BAG-1 were part of CeHsc70-containing complexes (Figure 3C). ADP, AMP-PNP and ATPγS also suppressed the binding of *BAG-1 to CeHsc70. This disruptive effect of nucleotides on BAG-1 binding implies that the function of BAG-1 as a nucleotide exchange factor for Hsc70 is conserved in the nematode system.

We tested the effect of BAG-1 on the hydrolysis activity of CeHsc70 and its truncation fragments. As shown for other eukaryotic systems [29], [38], addition of BAG-1 slightly stimulated the steady-state turnover rate of CeHsc70 (Table 2), but it dramatically reduced the rate in single-turnover assays (Table 2). Reduced single-turnover rates were obtained for all CeHsc70 truncation fragments, implying that the single-turnover condition of full nucleotide binding is not satisfied any more in the presence of BAG-1, likely due to a strongly reduced affinity for nucleotides (Table 2). In steady-state assays instead, the strongest stimulation was observed for CeHsc70-Δ512, while no stimulation was observed for CeHsc70-Δ545 (Table 2). Based on the known function of human Bag1 as NEF, it would be intuitive to find that the ATPase stimulatory effect on CeHsc70-Δ545 is the weakest. Likely, a CeHsc70-fragment, whose enzymatic turnover is not limited by nucleotide-release, may gain very little from the function of BAG-1.

**Table 2:** Enzymatic parameters of cofactor interactions with lid domain mutants.

	Single-turnover			Steady-state				
	k_hyd_	k_hyd, BAG-1_	k_hyd, DNJ-13_	k_cat_	k_cat, BAG-1_	K_D BAG-1_	k_cat, DNJ-13_	K_D, DNJ-13_
	(min^−1^)	(min^−1^)	(min^−1^)	(min^−1^)	(min^−1^)	(µM)	(min^−1^)	(µM)
CeHsc70	1.37±0.18	0.21±0.08	3.94±0.34	0.18±0.04	0.27±0.03	tight	0.43±0.06	tight
CeHsc70-Δ384	0.32±0.05	0.03±0.01	0.41±0.04	0.21±0.04	0.22±0.04	n.d.*	0.23±0.04	n.d.*
CeHsc70-Δ512	1.01±0.39	0.16±0.10	3.34±0.27	0.14±0.02	0.45±0.10	6.5±3.2	0.44±0.05	tight
CeHsc70-Δ545	0.08±0.03	0.02±0.03	14.38±2.06	0.09±0.02	0.14±0.03	n.d.*	0.58±0.10	4.2±2.2

ATPase activities were determined in standard buffer as described in the Materials and Methods section. DNJ-13 stimulation or BAG-1 inhibition were not observed in some experiments (denoted by “n.d.”). Consequently an apparent K_D_ cannot be deduced. The semi-quantitative value “tight” points to the fact that in the respective experiment, quantitative binding appeared substoichiometric. Consequently, no reasonable data fitting can be performed, using the normal absorption isotherm. K_D_ denotes the apparent affinity. The errors represent standard deviations of three independent experiments.

### DNJ-13 forms large ATP-dependent complexes with CeHsc70

**Figure 4:**
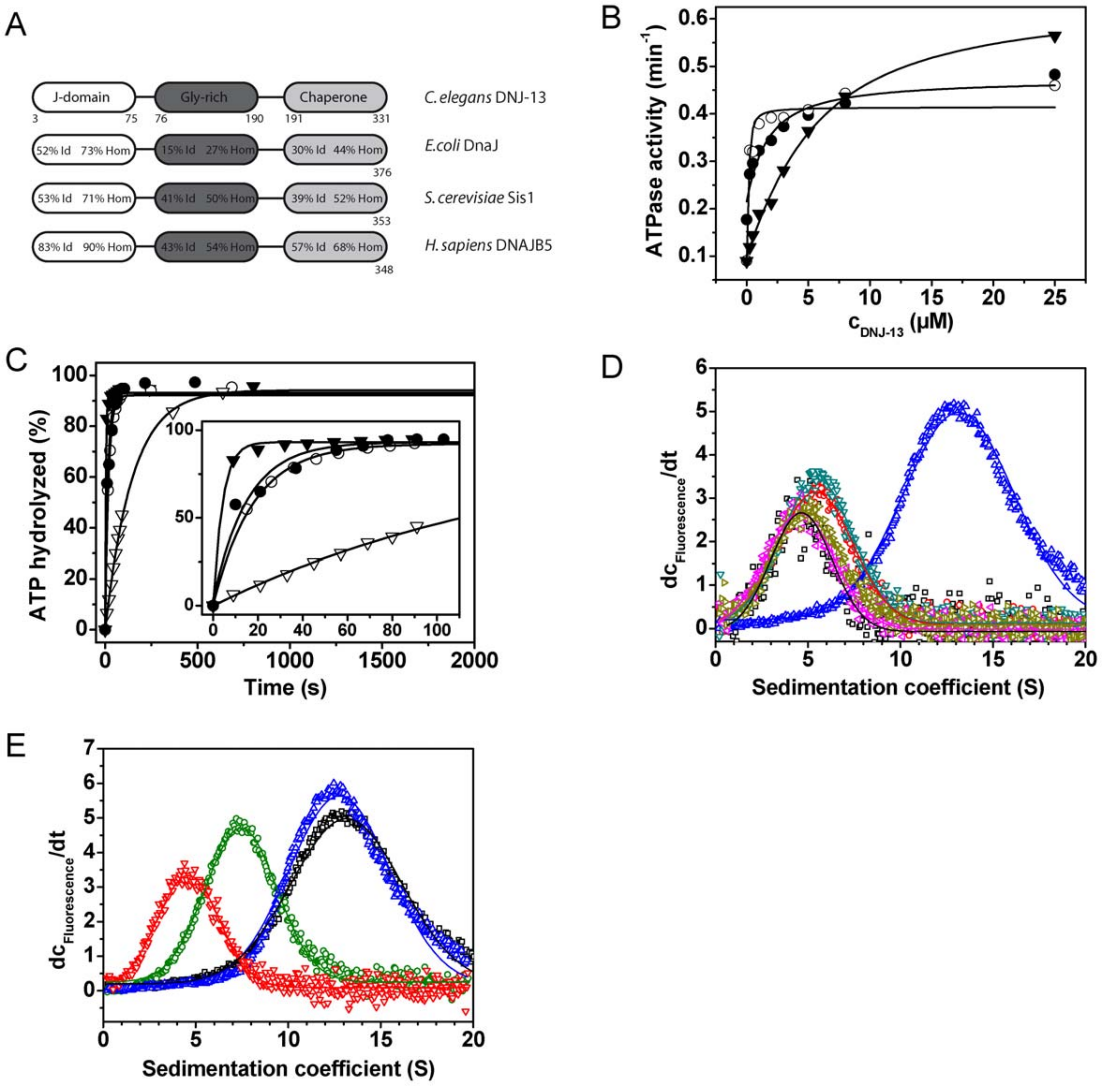
DNJ-13 interacts with CeHsc70 in presence of ATP and is released by BAG-1. (**A**) Domain organization of DNJ-13 homologs from *C. elegans*, *E. coli*, *S. cerevisiae* and *H. sapiens*. Percentages relate to identical (Id) and homolog (Hom) residues in respect to the nematode protein. (**B**) Steady-state ATPase activities were measured in the presence of increasing amounts of DNJ-13 for either CeHsc70 (•), CeHsc70-Δ512 (○) or CeHsc70-Δ545 (▾). Data were analyzed as described in the Materials and Methods section. (**C**) Single-turnover measurements of 10 µM CeHsc70-Δ384 (∇), CeHsc70-Δ512 (○), CeHsc70-Δ545 (▾) and CeHsc70 (•) in the presence of 15 µM DNJ-13. All data points were fit to single exponential functions. (**D**) dc/dt plots were generated from sedimentation velocity experiments of 300 nM *DNJ-13 in the absence (black) or in the presence of 3 µM CeHsc70 (pink). The influence of nucleotides was analyzed by addition of 4 mM of either ADP (gold), AMP-PNP (red), ATPγS (turqoise) or ATP (blue) to 300 nM *DNJ-13 and 3 µM CeHsc70. (**E**) dc/dt profiles of sedimentation velocity experiments of 300 nM *DNJ-13 in the presence of either 3 µM CeHsc70 (blue), CeHsc70-Δ384 (red), CeHsc70-Δ512 (black) or CeHsc70-Δ545 (green) in the presence of ATP.

The Hsp40-like protein DNJ-13 is the closest relative to yeast Sis1 and human DNAJB5 to be found in *C. elegans* (Figure 4A). We subcloned and purified recombinant DNJ-13. Steady-state ATPase measurements revealed that a twofold increase of the ATP turnover of CeHsc70 in the presence of DNJ-13 can be obtained. Also the lid domain truncations are stimulated by DNJ-13 (Figure 4B, Table 2), but not CeHsc70-Δ384. It is interesting to note, that CeHsc70-Δ545 shows the lowest apparent affinity (K_D,app_∼4 µM), while the interaction with CeHsc70-Δ512 is very strong, implying that the helical lid domain is not required for the interaction of the J-domain protein with CeHsc70.

In single-turnover experiments only a small increase in the hydrolysis rate of CeHsc70 and CeHsc70-Δ512 can be observed (Figure 4C, Table 2), despite the high affinity interaction (see Figure 4B, Table 2). Again, no stimulation is observed for CeHsc70-Δ384. Interestingly, CeHsc70-Δ545 which interacts most weakly with DNJ-13 is stimulated the most. This may be due to the shift of the rate-limiting step fully towards nucleotide-release in the presence DNJ-13 (Figure 4C, Table 2). Other variants in turn, which are limited by nucleotide-release interact strongly, but are barely stimulated.

In order to gain a deeper understanding of the mechanisms of cofactor regulation, we addressed the complex formation between DNJ-13 and CeHsc70 directly. We labeled DNJ-13 and subjected it to AUC. *DNJ-13 sedimented with a sedimentation coefficient of 4.0 S (Figure 4D) and the sedimentation and diffusion properties of a dimeric protein (s_20,w_ = 4.0 S±0.6 S; D_20,w_ = 5.67*10^−7^±1.13*10^−7^ m^2^ s^−1^; MW∼62 kDa), which is in agreement with DNJ-13 homologs from yeast [51] and bacteria [52]. The addition of CeHsc70 to *DNJ-13 did not change the sedimentation properties (Figure 4D) suggesting that no binding happens under these conditions. Also, addition of ADP or the non-hydrolysable ATP analogs AMP-PNP and ATPγS did not result in detectable interaction with CeHsc70. However, in the presence of ATP, CeHsc70 and *DNJ-13 formed a protein complex at 12 S (Figure 4D). It is important to note, that the molecular weight of a 12 S complex theoretically cannot be less than 210 kDa, but likely is larger. Thus, it can be assumed that in the presence of ATP, CeHsc70 binds to the *DNJ-13 dimer and forms a protein complex, which might be heterotetrameric consisting of two CeHsc70 and two DNJ-13 molecules (222 kDa). As the formation of the *DNJ-13•CeHsc70 complex was strictly dependent on the presence of ATP, DNJ-13 apparently specifically interacts with ATP-dependent conformations of CeHsc70, while other states are not recognized under these conditions. Analyzing the fragments of CeHsc70, we found CeHsc70-Δ384 to exhibit complex formation, whereas CeHsc70-Δ512 behaved similarly to CeHsc70. However, the binding of CeHsc70-Δ545 is weaker and potentially more dynamic as evident from the smaller s_20,w_ value of the protein complex at 7.5 S. Thus, these results support the ATPase assays, where CeHsc70-Δ545 also had shown a reduced apparent affinity for DNJ-13 (see Table 2).

### DNJ-13 and BAG-1 compete with each other's binding during the ATPase cycle

**Figure 5:**
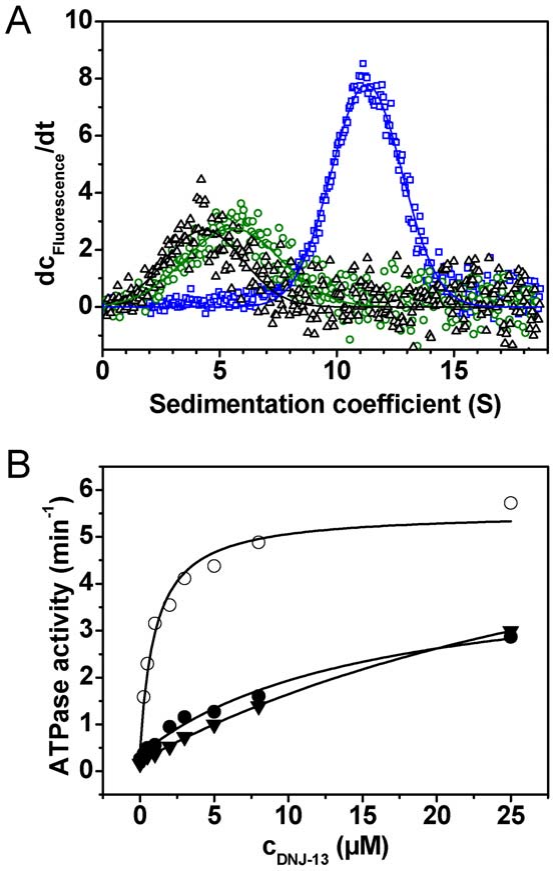
The ternary interaction of CeHsc70 with BAG-1 and DNJ-13 is affected by the lid domain truncations. (**A**) dc/dt plots were generated from sedimentation velocity experiments of 300 nM *DNJ-13 in the absence (black) or in the presence of 3 µM CeHsc70 and 4 mM ATP (blue). The influence of BAG-1 on complex formation was analyzed by addition of 15 µM BAG-1 to *DNJ-13-CeHsc70-ATP (green). (**B**) The ATPase activity of 1 µM CeHsc70 (•), CeHsc70-Δ512 (○) or CeHsc70-Δ545 (▾) was measured with increasing amounts of DNJ-13 in the presence of 2 µM BAG-1 in standard buffer at 25°C. Data analysis was performed as described in Materials and Methods.

It has been described for other Hsp70 systems that the interaction of Hsp40-like proteins and nucleotide exchange factors is competitive and results in strong stimulation of the ATPase activity [29], [30], [53], [54]. Indeed, upon addition of BAG-1 the large CeHsc70•*DNJ-13•ATP complex was not detectable anymore and only small species corresponding to mostly unbound *DNJ-13 could be seen (Figure 5A). Thus, BAG-1 has the ability to disrupt *DNJ-13•CeHsc70 complexes that are formed in the presence of ATP.

We also analyzed the ATPase activity of the CeHsc70-variants in the presence of both cofactors. We added DNJ-13 to CeHsc70 in the presence of 2 µM BAG-1 and observed a remarkable stimulation of ATP hydrolysis (Figure 5B). Nevertheless, in contrast to the stimulation in absence of BAG-1, now the apparent affinity is weak (K_D,app_ = 6.8 µM±3.2 µM), highlighting the competitive interaction between the two cochaperones. The interaction with CeHsc70-Δ545 appears even weaker exhibiting an almost linear increase during titration up to 25 µM DNJ-13 (Figure 5B). Only for CeHsc70-Δ512•BAG-1 complexes, a saturation curve could be observed (K_D,app_ = 1.2 µM±0.4 µM), confirming the high apparent affinity of this truncation variant for DNJ-13. Thus, also in these assays the truncation mutants CeHsc70-Δ545 and CeHsc70-Δ512 behaved differently and in analogy to the analysis of the binary complexes (see Figure 4B and 4D), DNJ-13 binds more strongly to CeHsc70-Δ512 compared to CeHsc70-Δ545.

### Substrate refolding by CeHsc70 requires optimal concentrations of BAG-1 and DNJ-13

**Figure 6:**
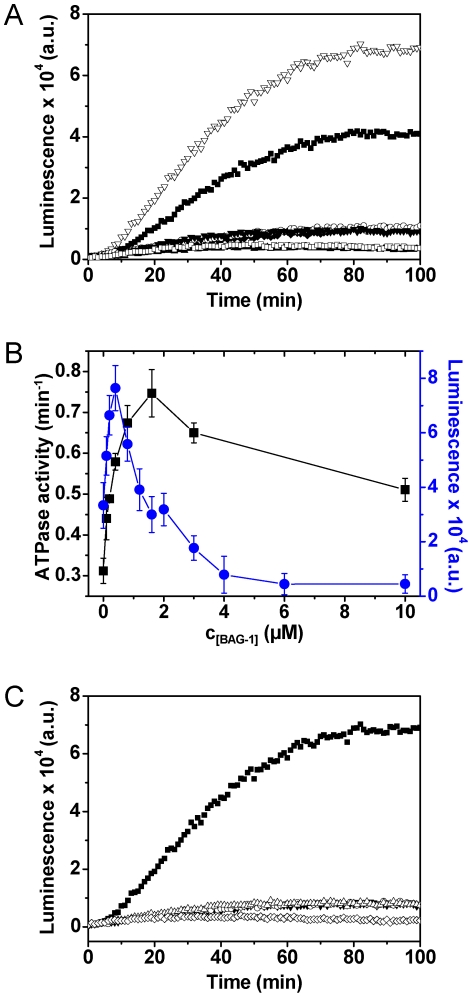
Lid domain truncations reduce the refolding ability of CeHsc70. (**A**) Kinetics of firefly luciferase refolding in the presence of different chaperone combinations: CeHsc70/DNJ-13/BAG-1 (∇), CeHsc70/DNJ-13 (▪), CeHsc70/BAG-1 (♦), CeHsc70 (○), BAG-1 (□) and DNJ-13 (▾). Additionally the luminescence of a sample without chaperones and cofactors was analyzed (▴). Protein concentrations were 3.2 µM CeHsc70, 0.8 µM DNJ-13 and 0.4 µM BAG-1. Luciferase refolding assays were carried out as described in Materials and Methods. (**B**) Steady-state ATPase activities (black squares, left ordinate) and luciferase refolding efficiency (blue circles, right ordinate) were determined for 3.2 µM CeHsc70 and 0.8 µM DNJ-13 at different BAG-1 concentrations under standard conditions. (**C**) The luciferase refolding activity of either CeHsc70 (▪), CeHsc70-Δ545 (▾), CeHsc70-Δ512 (○) or CeHsc70-Δ384 (Δ) was determined in the presence of DNJ-13 and BAG-1. Additionally a control without chaperones and cofactors (◊) was recorded.

It is not fully understood, how the two cochaperones contribute to the folding activity of Hsc70 in the eukaryotic system. In particular, nucleotide exchange factors had been found to have both supportive and inhibitory functions in eukaryotes [35]–[38], [55]. We analyzed the refolding activity of CeHsc70 on denatured luciferase in the absence and presence of DNJ-13 and BAG-1. CeHsc70 alone was not able to refold luciferase, while addition of DNJ-13 resulted in refolding activity (Figure 6A). Addition of substoichiometric amounts of BAG-1 increased the refolding efficiency further (Figure 6A and 6B), but higher concentrations of BAG-1 reduced it to baseline levels (Figure 6B) revealing a clear optimum of NEF concentrations similar to the prokaryotic system [56]. We were interested, whether ATP hydrolysis followed the same trend. The efficiency of luciferase refolding does not correspond to ATPase activities measured under identical conditions, implying that these two processes – optimal folding activity and maximal ATP hydrolysis – are independent and do not share the same cochaperone requirements (Figure 6B). Interestingly though, the positive influence of BAG-1 on the hydrolysis rate vanishes at high concentrations, suggesting that in ATPase assays also a competitive inhibition of the system may become observable.

Having shown that the truncations in the lid domain do not prevent ATP hydrolysis and interaction with CeHsc70 cofactors, we aimed at elucidating the influence of these deletions on the protein folding activity. Under neither concentration of cofactors, we were able to regain luciferase activity above the baseline level (Figure 6C), implying that in similarity to the human system [22], [57] the presence of the lid domain, while not essential for hydrolysis and cofactor interactions, is required for the functional activity of the Hsc70 chaperone machinery from *Caenorhabditis elegans*.

## Discussion

### Regulation of the CeHsc70 ATPase cycle by the helical lid domain

**Figure 7:**
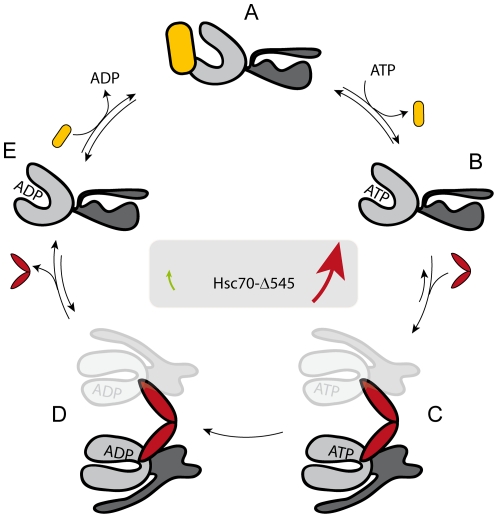
A model for the regulation of CeHsc70's ATPase by the lid domain. A structural hypothesis for the regulation of the CeHsc70 ATPase cycle may be formulated based on the structures 2KHO of DnaK (*74*) and 3D2E of the Hsp70-homolog protein Sse1 (*75*). After initial binding of ATP to the NBD of CeHsc70 (Step A→B), conformational changes result in a hydrolysis competent conformation (Step B→C). This reaction is favored in CeHsc70, as evident from the observation, that hydrolysis is not rate-limiting. The helical lid likely regulates the equilibrium or the kinetics of the B→C transition, as this reaction appears to be much slower in CeHsc70-Δ545. DNJ-13 (red) accelerates the formation of the hydrolysis-competent conformation and thus promotes ATP hydrolysis. ATP hydrolysis likely is irreversible (Step C→D). After hydrolysis, DNJ-13 leaves the complex and Hsc70 returns to its open conformation (Step D→E). BAG-1 (yellow) acts to displace the nucleotide (Step E→A). Based on this model, simultaneous BAG-1 and DNJ-13 binding to CeHsc70 would be mutually exclusive, although several intermediate steps might exist during this sophisticated cycle.

We analyzed the Hsc70 system of *C. elegans* by utilizing truncation mutants in the C-terminal lid domain. It is evident from our data that all domains participate during the hydrolysis reaction: The isolated ATPase domain (CeHsc70-Δ384) can hydrolyze ATP, but more C-terminal regions stimulate the intrinsic hydrolysis reaction. As studies on other model organisms suggest, conformational changes in CeHsc70 lead to a hydrolysis-competent state. The initial segment of the lid-domain can apparently inhibit this process by slowing down these pre-hydrolysis conformational changes (Figure 7). Structures at the C-terminus of the lid domain (amino acid 545 to 640) obviously reduce this inhibitory effect, giving the helical lid inhibitory as well as reactivating parts. Previous studies reported that ATP binding induces conformational changes in the lid leading to its displacement away from the peptide binding site [58], [59]. Based on our data, we can establish a model (Figure 7), in which lid truncation mutants can shift the conformational equilibrium either towards the hydrolysis competent state (CeHsc70-Δ512) or towards the pre-hydrolysis state (CeHsc70-Δ545). CeHsc70 apparently more closely resembles CeHsc70-Δ512, as its turnover also is limited by nucleotide release. While it cannot be excluded that parts of the truncated lid domain imitate a bound substrate protein in CeHsc70-Δ545 [60], [61], the reduced hydrolysis rate, however, renders this scenario unlikely. Usually Hsp70 hydrolysis is stimulated in the presence of client proteins [62], [63]. Interestingly, intermediate lid-truncations in DnaK, corresponding to CeHsc70-Δ545, showed a 2–8 fold activation of steady-state hydrolysis, suggesting that in this case the lid indeed may have served as an internal substrate [64], while the almost lid-free truncation only showed slight effects [65].

### Association of the cofactors DNJ-13 and BAG-1 with CeHsc70

It is interesting to see, that also the binding of the Hsc70 cofactors BAG-1 and DNJ-13 is influenced by lid deletions. Clearly, in CeHsc70 the lid domain is not required to bind DNJ-13 or BAG-1. As observed for the bacterial system [66], [67], CeHsc70-Δ512 can be stimulated by its J-protein DNJ-13. Thus, it is likely that the changes in cofactor affinities result from alterations to the conformational cycle and the rate-limiting step, which are due to the deletions in the lid domain.

DNJ-13 binds more weakly to the “open” CeHsc70-Δ545 than to the hydrolysis-competent CeHsc70-Δ512 or CeHsc70. It is intriguing that the conformational changes in response to ATP-binding provide the platform for the high-affinity interaction with DNJ-13. In similarity to the ATP hydrolysis reaction itself, the initial segment of the lid domain acts as an inhibitor of the CeHsc70•DNJ-13 interaction. The competitive binding of BAG-1 and DNJ-13 suggests that competition is generated by favoring a specific conformation during the hydrolysis cycle, which excludes or reduces the apparent affinity of the other cofactor. As such, the presence of BAG-1 weakens the apparent binding constant of DNJ-13 (see Figure 5B). It is generally interesting to note that the strongest effects on ATP turnover occur during the weak cofactor interactions. This is intuitive, as for a productive acceleration of the hydrolysis reaction an unfavorable conformational transition has to be overcome by cofactor binding.

### Conservation of the Hsc70 system in nematodes

A large number of studies exist on the hydrolysis reaction of Hsc70 proteins from other model organisms and the regulation of their activity by substrate proteins and cofactors. In particular, the DnaK-system of *E. coli* has been characterized in considerable detail. Several mutations in DnaJ and DnaK have been described, which disrupt the binding of cofactors and a mechanism of the interaction had been postulated that explains the stimulation of the ATPase rate of DnaK in the presence of DnaJ [25], [66]–[79]. Substrate-lid truncations in DnaK have been characterized and revealed effects on substrate binding and refolding activities, but only weak effects on ATP-hydrolysis [47], [64], [65]. The inhibitory properties of the lid domain, as observed for CeHsc70-Δ545, have not been uncovered in these studies. It is important to note that strong differences exist between DnaK and the eukaryotic proteins, specifically within the helical lid domain, which is almost unrelated in terms of primary sequence. The function of the lid domain as an inhibitor of the intrinsic hydrolysis rate and thus the potential coupling of its motions to the hydrolysis reaction might hence be different in the bacterial system [47], [80]. Fewer data are available for eukaryotic systems. In yeast, the very low hydrolysis rates of Ssa1 and Ssa2 render comparison to the nematode system difficult [43]. The best eukaryotic match might be the mammalian system, but no systematic analysis of lid truncations has been performed here yet. As a consequence, it remains to be determined, whether the effects observed in our study are of general importance to all Hsp70 systems or whether they represent a specialty of *C. elegans*. Our data comparing the activity and stability of the human and nematodal versions of Hsc70 point to the fact that the slightly higher basal activity of CeHsc70 at equal temperatures may be due to a shifted activity and stability optimum that coincides surprisingly well to the optimum growth or body temperature of both organisms.

Also, regarding the interaction between Hsc70 and Hsp40 a wealth of data exists. The strict dependence of the Hsc70/J-protein interaction on the presence of ATP has been observed in studies using Hsp70-systems from bacteria, eukaryotes and organelles [18], [74], [75], [77], [81], [82]. However, recent data on the ER-resident Hsp70-system highlight that for some systems complex formation is also possible in the presence of ADP [83] and consequently the regulation may be more complex. Also, DnaJ•DnaK complexes have been observed in the presence of ADP during NMR experiments [77]. For the *C. elegans* system, we observe complex formation only in the presence of ATP, but based on the fast ATP hydrolysis rates, it has to be assumed that in the observed assemblies hydrolysis has taken place and the interaction also may happen as a post-hydrolysis DNJ-13•CeHsc70•Mg-ADP-P_i_ complex. As AUC only provides very limited kinetic information, the dissociation rate of this complex cannot be determined. Still, it is unlikely that the complex is assembled at all stages of the ATPase cycle, suggesting that the nucleotide-release controlled steady-state hydrolysis rate of 0.43 min^−1^ to 0.58 min^−1^ (Table 2) serves as an upper limit for the complex stability.

It is surprising that large DNJ-13•CeHsc70•Mg-ADP-P_i_ complexes are formed during AUC. As Hsp40-like proteins contain dimerization sequences at the C-terminus, the formation of these assemblies as heterotetrameric complexes appears possible. Certainly, it cannot be ruled out that a combination of specific and unspecific interactions leads to the formation of these assemblies [84]. Given the high concentration of CeHsc70 and the presence of substoichiometric amounts of DNJ-13 in the luciferase-refolding assays, it is also possible that this multimeric protein complex may serve as a functional species in the refolding of firefly luciferase.

## Materials and Methods

### Worm handling and analysis of the heat-shock response

Worms were handled according to standard procedures and grown on NGM plates seeded with OP50 bacteria. To analyze the heat-shock response worms were synchronized and grown for four days on NGM plates at 20°C to obtain young adult worms (YA stage). Plates containing on average 100 nematodes were sealed in plastic bags and heat-shocked at different temperatures in a water bath for two hours. Plates were removed from the plastic bags and returned to the 20°C incubator. After 12 hours the GFP expression was localized and quantified by visual inspection. “100% induction” required bright expression in all nematodes on the plate in the following cells: pharyngeal muscle cells, intestinal rings 1, 8 and 9, both spermathecae, body wall muscle cells and a visible induction in hypodermal cells. Incomplete induction patterns or heterogeneity between individual worms was evaluated by intermediate %-values. Survival was scored based on the recovery of nematodes from the heat-shock after 24 hours. The experiment was repeated three times. The strain containing the integrated *hsp-70::GFP* construct was a kind gift of Richard I. Morimoto (Northwestern University, Evanston, IL, USA).

### Sequence alignments and determination of homologies

The domain boundaries were defined according to the Conserved Domain Database after a conserved domain query on the protein sequence (http://blast.ncbi.nlm.nih.gov/). In order to determine the degree of conservation within one domain, identical and homologous residues, as identified by the BLAST alignment tool, were determined and percentage values for each domain were calculated.

### Expression clones and protein purification

Expression plasmids for His_6_-fused CeHsc70 fragments were generated based on the pET28a (Merck KGaA, Darmstadt, Germany) plasmid as described earlier [14]. The coding sequence of *dnj-13* was cloned into the pET28a vector for protein expression using a full-length cDNA clone of *dnj-13* in the RNAi plasmid L4440 (Thermo Scientific, Huntsville, AL, USA) as a template. To clone *bag-1*, a cDNA preparation of *C. elegans* nematodes was generated, using the Qiagen RNAeasy kit (Qiagen, Hilden, Germany) and reverse transcriptase (Promega, Madison, WI, USA) with a polyT-primer according to the manufacturer's protocol.

Proteins were expressed in the *E. coli* BL21-CodonPlus (DE3)-RIL strain (Agilent Technologies, Santa Clara, CA, USA). Bacteria were grown to an OD_600_ of 0.8 and expression was induced with 1 mM IPTG. After four hours, bacterial cells were harvested and resuspended in 40 mM HEPES/KOH, pH 7.5, 300 mM KCl. Bacterial cells were lysed using the cell disruption instrument TS 0.75 (Constant Systems Ltd., Northants, UK) and the soluble fraction was applied to a HisTrap 5 ml column (GE Healthcare, Chalfont St Giles, UK). The protein was eluted with disruption buffer containing 300 mM imidazole. CeHsc70 and BAG-1 were further purified using ResourceQ ion exchange chromatography and size exclusion chromatography on a Superdex 75 HiLoad column (both GE Healthcare, Chalfont St Giles, UK). DNJ-13 was loaded onto a ResourceS ion exchange column and was further purified by size exclusion chromatography using a Superdex 75 HiLoad column (both GE Healthcare, Chalfont St Giles, UK). HsHsc70 was expressed as described previously [85] and purified on DEAE-Sepharose, Resource Q (both GE Healthcare, Chalfont St Giles, UK), Fluoroapatite (Bio-Rad, Hercules, CA, USA) and for polishing on Superdex 200 HiLoad (GE Healthcare, Chalfont St Giles, UK) columns. The purity of the proteins was determined to be more than 95% according to SDS-PAGE. Protein concentrations were 220 µM for BAG-1, 160 µM for DNJ-13 and 60 µM for CeHsc70. The His_6_ tag was removed by digestion of 1 mg of His_6_-CeHsc70 with 5 U of thrombine (Merck KGaA, Darmstadt, Germany) according to the manufacturer's instructions and subsequent purification via a HisTrap 1 ml column (GE Healthcare, Chalfont St Giles, UK, see above). The removal of the tag did not affect the stability of CeHsc70 and its activity was increased within the range of error (compare Figure S2).

### Differential scanning fluorimetry

Differential scanning fluorimetry (DSF) was deployed to determine the unfolding temperature of CeHsc70, its fragments and HsHsc70 by monitoring an increase in the fluorescence of SYPRO orange upon binding to exposed hydrophobic parts of the protein [86]. The dye (Invitrogen, Carlsbad, CA, USA), is diluted 1∶100 in buffer solution (40 mM HEPES/KOH pH 7.5, 150 mM KCl). This pre-mix is diluted again 1∶10 in buffer solution containing 0.5 mg/ml CeHsc70. Temperature dependent unfolding of CeHsc70 and the resulting increase in fluorescence was measured in the Mx3000P qPCR System (Agilent Technologies, Santa Clara, CA, USA). Fluorescence reads were performed at a heating rate of 0.5°C/min every minute. All measurements were performed in triplicates. Melting curves were normalized and averaged. ADP was added to 2 mM where indicated.

### Circular dichroism thermal transitions

Circular dichroism (CD) temperature transitions were recorded in 40 mM HEPES, pH7,5, 150 mM KCl at 217 nm. The heating rate was 0.5°C/min, starting at 12°C. Moving averaging at a window size of 5 datapoints was applied to the curves to reduce noise. The curves were normalized to allow comparison. The data were not fitted to obtain thermodynamic unfolding parameters, as transitions were irreversible.

### Steady-state ATPase activity measurements

Steady-state ATPase activities were determined as described earlier [14], [87]. In short, an ATP-regenerating system was used, employing lactate dehydrogenase, NADH, phosphoenol pyruvate and pyruvate kinase in a buffer containing 40 mM HEPES/KOH, pH 7.5, 150 mM KCl, 5 mM MgCl_2_. Assays were started by addition of 2 mM ATP, the assay temperature was 25°C for all experiments, when not indicated differently.

The influence of cofactors on the CeHsc70 activity was analyzed by titration. The ATPase activities at different cofactor concentrations were fit to obtain apparent K_D_-values according to the following equation:
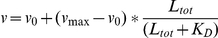
In cases where the apparent affinity of the interaction was so high that stoichiometric or substoichiometric binding was observed, the following equation was used:
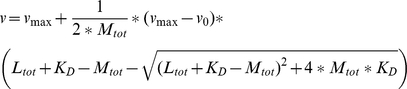



### Single-turnover ATPase activity experiments

Single-turnover ATPase assays were based on the separation of [α-^32^P]-ADP from [α-^32^P]-ATP (Hartmann Analytic, Braunschweig, Germany) by thin layer chromatography [88]. 30 µl of a solution containing 20 µM CeHsc70 and 30 µM DNJ-13 or BAG-1 in assay buffer (40 mM HEPES/KOH pH 7.5, 150 mM KCl, 5 mM MgCl_2_) were mixed to a final concentration of 5 µM ATP containing 1.0 µCi [α-^32^P]-ATP. At defined time points, aliquots of 3 µl were withdrawn from the assay reaction and added to 2 µl 100 mM EDTA, pH 8.0, to stop the hydrolysis reaction. 0.9 µl of these aliquots were applied to polyethylenimine-cellulose plates (Merck Bioscience, Darmstadt, Germany) and chromatographically separated using a mobile phase of 0.5 M LiCl in 2 N formic acid. Evaluation of the chromatogram was performed using a Typhoon 9200 Variable Mode Imager (GE Healthcare, Chalfont St Giles, UK). Spot intensities were determined using ImageQuant (GE Healthcare, Chalfont St Giles, UK). The values were normalized and plotted against the reaction time. Data analysis was performed using single-exponential functions for all assays.

### Analytical ultracentrifugation with fluorescently labeled cofactors

Analytical ultracentrifugation (AUC) was performed using fluorescently labeled BAG-1 (*BAG-1) and DNJ-13 (*DNJ-13). BAG-1 was labeled at its sole cysteine residue at amino acid position 7 with Alexa Fluor 488 C_5_-maleimide (Invitrogen, Carlsbad, CA, USA). DNJ-13 was labeled with 5-(and-6)-carboxyfluorescein succinimidylester (Invitrogen, Carlsbad, CA, USA) at neutral pH in order to obtain preferential labeling at the N-terminal amine group. Both labels were added to the protein (1 mg/ml) at a threefold molar excess upon continuos mixing. After 2 h of incubation at room temperature, unreacted label was quenched by adding DTT to a final concentration of 20 mM in the case of *BAG-1 or an Tris to a final concentration of 100 mM in the case of *DNJ-13. Free label was separated from the labeled protein by size-exclusion chromatography. The labeling efficiency of *BAG-1 and *DNJ-13 was determined using the manufacturer's guidelines and was found to be 0.95 and 1.2, respectively.

AUC was performed with a ProteomeLab XL-A ultracentrifuge (Beckman Coulter, Brea, CA, USA) equipped with a fluorescence detection system (Aviv Biomedical, Lakewood, NJ, USA). The centrifugation experiments in general were performed at 20°C at 42 000 rpm. Labeled protein at a concentration of 300 nM was sedimented in the absence and presence of binding partners and different nucleotides. Sedimentation velocity experiments were evaluated using dc/dt analysis as described before [89]–[91]. Species distributions in dc/dt plots were fit to Gaussian or bi-Gaussian functions in order to obtain the s_20,w_ values of the observed sedimentation boundaries. It is important to note, that in particular when binding affinities are low and the interaction is dynamic, sedimentation boundary analysis, as employed by the dc/dt-approach, can result in a reduced s_20,w_ value compared to an irreversible protein complex of the same composition. Full analysis of sedimentation runs, as performed for *BAG-1 and *DNJ-13 in the absence of additional factors, was performed using the Finite Element Whole Boundary Fitting and C(s) methods of the UltraScan software package [92], which fits the data set assuming one species of particles and determines the sedimentation coefficient s_20,w_ and the Diffusion coefficient D_20,w_. These values are then used to obtain the molecular weight of the sedimenting particle. The molecular weight, s_20,w_ and D_20,w_ of *DNJ-13 and *BAG-1 corresponded to the values obtained for the unlabeled proteins.

### Luciferase refolding assay

Recombinant luciferase (10 µM) was denatured for 45 min at room temperature in denaturing buffer (25 mM HEPES/NaOH, pH 7.5, 50 mM KCl, 15 mM MgCl2, 1 mM ATP, 10 mM DTE, 0.05 mg/mL BSA, 5 M GdmCl). For refolding, denatured luciferase was diluted 1∶125 in luminescence buffer (25 mM HEPES/NaOH, pH 7.5, 50 mM KCl, 15 mM MgCl_2_, 1 mM ATP, 2 mM DTE, 0.05 mg/mL BSA, 240 µM CoA, 0.1 mM luciferin, 10 mM PEP, 50 µg/mL pyruvate kinase) containing 3.2 µM CeHsc70, 0.8 µM DNJ-13 and 0.4 µM BAG-1. Reactions were carried out in white 96-well LIA-plates (Greiner Bio-One, Solingen, Germany). Luciferase activity was detected continuously over a time period of 2 h at 25°C by using a Tecan GENios™ microplate reader (Tecan Trading Ltd., Männedorf, Switzerland).

### Limited proteolysis

The CeHsc70 fragments Δ512, Δ545 and the full-length protein were digested by chymotrypsin at 25°C. The reaction was carried out in 40 mM HEPES/KOH, pH 7.5, 20 mM KCl, 10 mM CaCl_2_ with a final concentration of 20 µg/ml α-chymotrypsin (Sigma-Aldrich, St. Louis, MO, USA) and 600 µg/ml of the corresponding proteins. By adding PMSF, dissolved in DMSO to a final concentration of 33 mM at the indicated time points, the digestion was stopped. The samples were immediately boiled in 1× loading buffer, resolved electrophoretically on 12% polyacrylamide gels and stained with coomassie blue according to standard protocols.

### Statistical validation

*In vivo* experiments were replicated three times. The results at each temperature were averaged among these replicates and the standard deviation was calculated. Both values are presented in the respective figures. CD unfolding transitions, single-turnover ATPase, AUC and luciferase refolding assays were performed in three separate experiments and representative data are shown. In cases, where kinetic parameters were derived from these assays, the values obtained from fitting the three independent kinetics were averaged and are given together with their respective standard deviation. Steady-state ATPase measurements were also performed in triplicates and the obtained k_cat_ and apparent K_D_ values and their standard deviation were obtained by averaging.

## Supporting Information

Figure S1 **Thermal stability of CeHsc70 versus HsHsc70.** DSF melting curves indicate that Hsc70 from *C. elegans* (□) is about 10°C less stable than the human ortholog (○). Adding ADP stabilized CeHsc70 (▪) as well as human Hsc70 (•) to a similar extent. Error bars reflect the standard deviation of three experiments.
(TIF)

Figure S2 **The influence of a His_6_ tag on ATPase activity.** His_6_-CeHsc70 as used throughout the study and as described in the Materials and Methods section was compared to a His_6_ free protein batch, generated from the same stock. The removal of the tag increases the average activity within the margin of error (standard deviation of three measurements).
(TIF)

Figure S3 **Comparative stabiliy of CeHsc70 truncations.** (**A**) CD thermal transitions indicate that all variants of CeHsc70 - although about 10°C less stable than the human protein (grey) are comparably stable (CeHsc70, yellow; CeHsc70-Δ545, light blue; CeHsc70-Δ512, red; CeHsc70-Δ384, green). Compare Table 1 for transition midpoints. (**B**) A similar stability for all fragments is also highlighted by limited proteolysis. CeHsc70, CeHsc70-Δ545, and CeHsc70-Δ512 were subjected to α-chymotrypsin digestion and subsequent denaturing gel electrophoresis after quenching the reaction at the indicated timepoints. The kinetics are similar for all proteins, which all degrade to a species indicated by the asterisk. This implies that the overall structure of the core domain is preserved. (**C**) DSF further confirms a comparable overall CeHsc70 (□), CeHsc70-Δ545 (Δ), and CeHsc70-Δ512 (○) the fragments and the wild type proteins are stabilized in a highly similar manner by the addition of ADP (▪, ▴, •, respectively; see Table 2 for transition midpoints). (**D**) CeHsc70-Δ545 (□) exhibits a slightly different transition curve. Yet, the transition midpoint at about 37°C is comparable to the other fragments. The stabilization of the structure by roughly 10°C through the addition of ADP (▪) is also observed.
(TIF)
