# Supplementary figures and images for: The Lid Domain of Caenorhabditis elegans Hsc70 Influences ATP Turnover, Cofactor Binding and Protein Folding Activity
Source: PLoS One. 2012 Mar 29;7(3):e33980. doi: 10.1371/journal.pone.0033980 (PMC3315512; doi:10.1371/journal.pone.0033980)

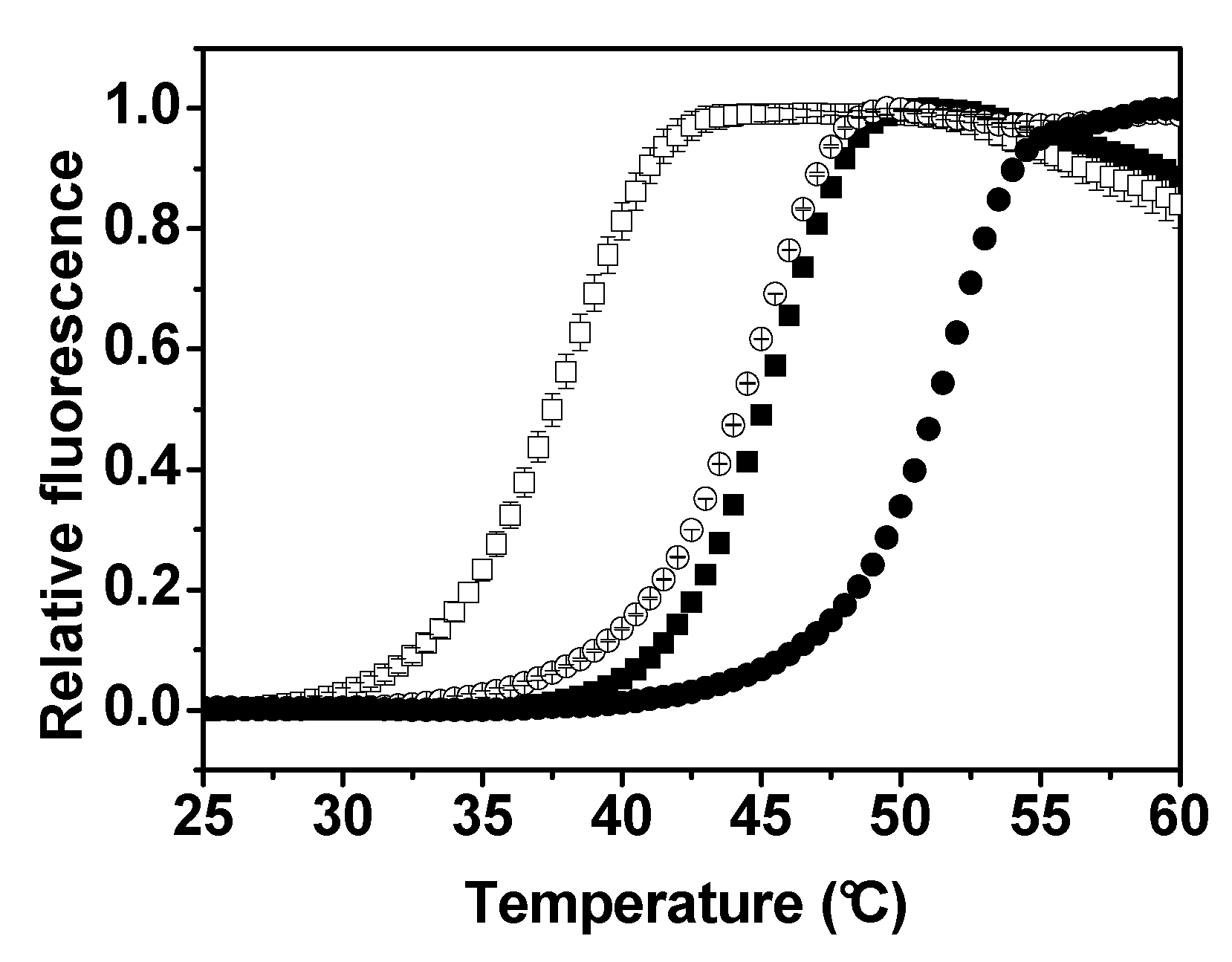

Supplement: Figure S1 — Thermal stability of CeHsc70 versus HsHsc70. DSF melting curves indicate that Hsc70 from C. elegans (□) is about 10°C less stable than the human ortholog (○). Adding ADP stabilized CeHsc70 (▪) as well as human Hsc70 (•) to a similar extent. Error bars reflect the standard deviation of three experiments. (TIF) [file pone.0033980.s001.tif]

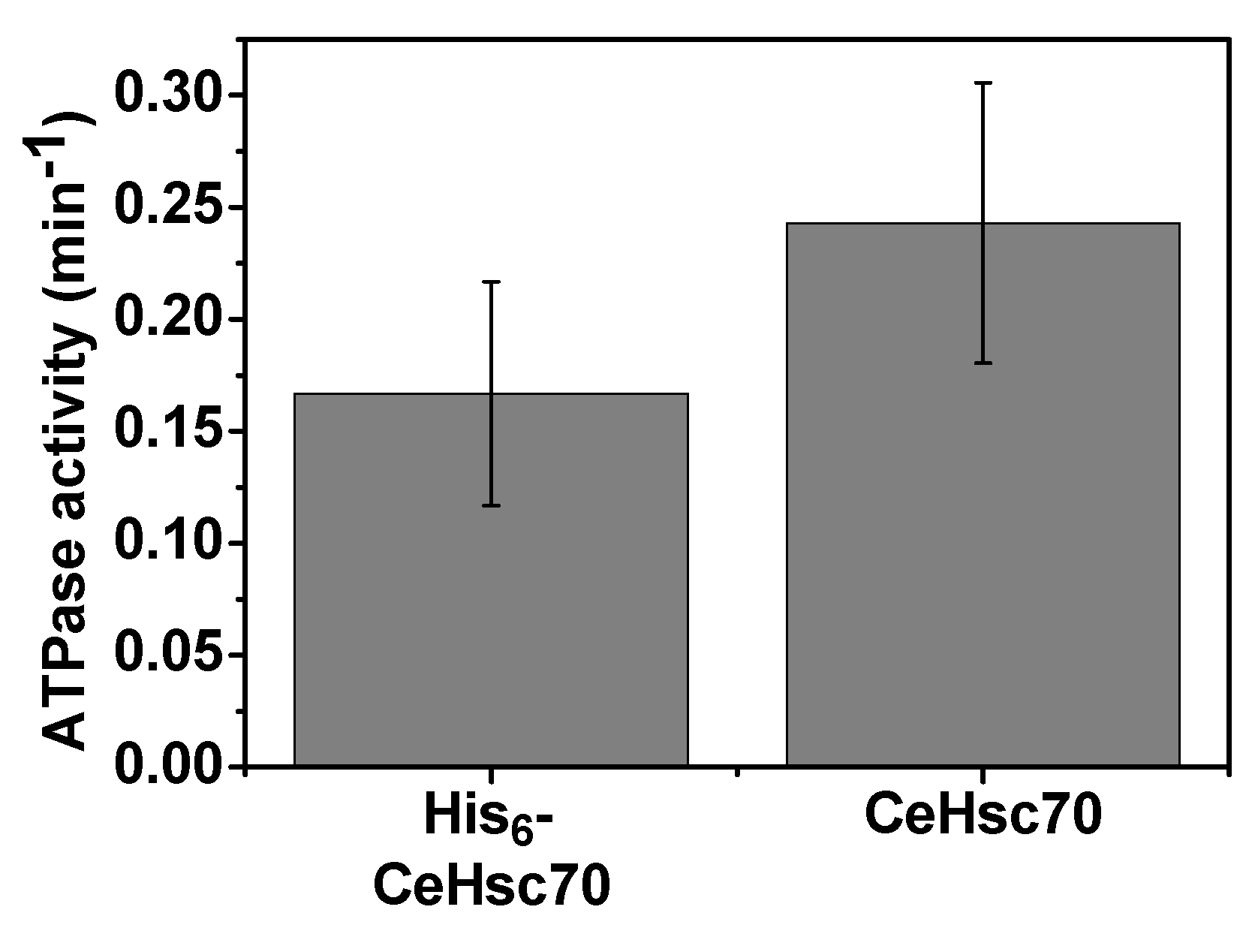

Supplement: Figure S2 — The influence of a His6 tag on ATPase activity. His6-CeHsc70 as used throughout the study and as described in the Materials and Methods section was compared to a His6 free protein batch, generated from the same stock. The removal of the tag increases the average activity within the margin of error (standard deviation of three measurements). (TIF) [file pone.0033980.s002.tif]

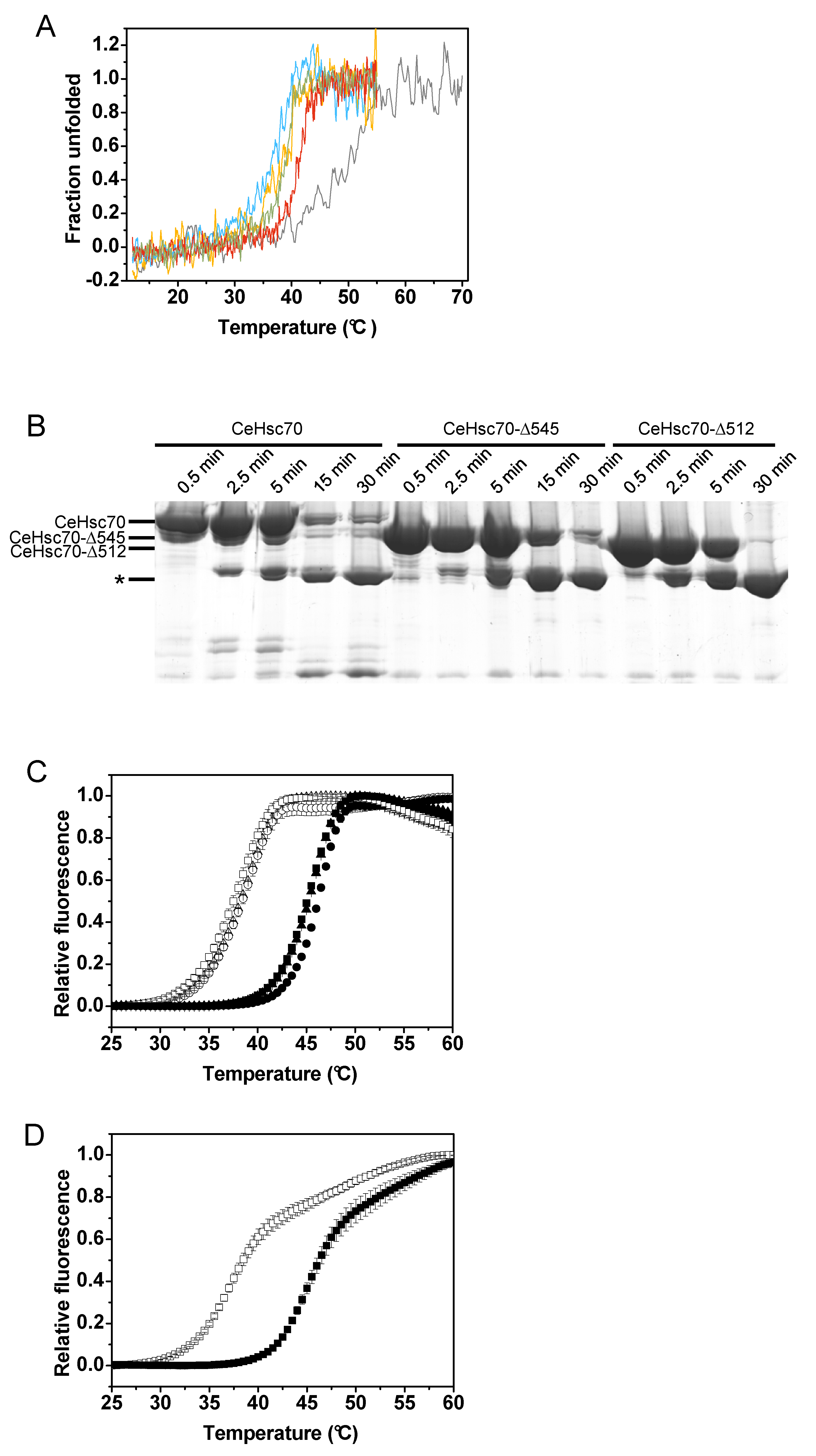

Supplement: Figure S3 — Comparative stabiliy of CeHsc70 truncations. (A) CD thermal transitions indicate that all variants of CeHsc70 - although about 10°C less stable than the human protein (grey) are comparably stable (CeHsc70, yellow; CeHsc70-Δ545, light blue; CeHsc70-Δ512, red; CeHsc70-Δ384, green). Compare Table 1 for transition midpoints. (B) A similar stability for all fragments is also highlighted by limited proteolysis. CeHsc70, CeHsc70-Δ545, and CeHsc70-Δ512 were subjected to α-chymotrypsin digestion and subsequent denaturing gel electrophoresis after quenching the reaction at the indicated timepoints. The kinetics are similar for all proteins, which all degrade to a species indicated by the asterisk. This implies that the overall structure of the core domain is preserved. (C) DSF further confirms a comparable overall CeHsc70 (□), CeHsc70-Δ545 (Δ), and CeHsc70-Δ512 (○) the fragments and the wild type proteins are stabilized in a highly similar manner by the addition of ADP (▪, ▴, •, respectively; see Table 2 for transition midpoints). (D) CeHsc70-Δ545 (□) exhibits a slightly different transition curve. Yet, the transition midpoint at about 37°C is comparable to the other fragments. The stabilization of the structure by roughly 10°C through the addition of ADP (▪) is also observed. (TIF) [file pone.0033980.s003.tif]
